# Glutathione-S-Transferase *p*1 Gene Promoter Methylation in Cell-Free DNA as a Diagnostic and Prognostic Tool for Prostate Cancer: A Systematic Review and Meta-Analysis

**DOI:** 10.1155/2023/7279243

**Published:** 2023-01-28

**Authors:** Jinghe Ye, Mao Wu, Long He, Peng Chen, Hongtao Liu, Hongwei Yang

**Affiliations:** ^1^Department of Graduate School, China Medical University, Shenyang, China; ^2^Department of Organ Transplantation Center, General Hospital of Northern Theatre Command, Shenyang, China; ^3^Department of Urology, General Hospital of Northern Theatre Command, Shenyang, China

## Abstract

**Background:**

Promoter methylation of glutathione-S-transferase *p*1 (GSTP1) is related to the occurrence of prostate cancer (PCa), but reports are inconsistent about the accuracy of GSTP1 promoter methylation in PCa diagnosis and prognosis. Therefore, we systematically evaluated the diagnostic and prognostic value of GSTP1 promoter methylation in PCa.

**Methods:**

The PubMed, EMBASE, Web of Science, and PMC databases were searched for all relevant studies from the date of inception to November 31, 2021. We compared differences in the incidence of GSTP1 promoter methylation in cfDNA between prostate cancer patients and controls. The odds ratio (OR) and hazard ratio (HR) were used as effect sizes, and the result of each effect size is expressed as a 95% confidence interval (95% CI).

**Results:**

Our meta-analysis showed that the combined sensitivity and specificity of GSTP1 promoter methylation in cfDNA for the diagnosis of prostate cancer were 0.37 (95% CI = 0.23, 0.53) and 0.97 (95% CI = 0.88, 0.99), respectively. The area under the curve (AUC) with 95% CI was 0.78 (95% CI = 0.75, 0.82). For prognostic variables, hypermethylation of GSTP1 was associated with shorter survival in PCa (HR = 2.57, 95% CI = 1.30, 5.10), with statistical significance in between-study heterogeneity (*I*^2^ = 72%, *P*=0.006). The results of the subgroup analysis indicated that the heterogeneity of studies may be due to differences in the observed indicators.

**Conclusions:**

The results of the meta-analysis substantiate the high specificity of promoter methylation of GSTP1 in cfDNA for the diagnosis of prostate cancer, and it may be used to more precisely evaluate the prognosis of patients with prostate cancer. It may be helpful for the early detection of prostate cancer, but it still must be combined with traditional prostate-specific antigen (PSA) or other methylated genes to accomplish this goal.

## 1. Introduction

Prostate cancer (PCa) has shown a high incidence worldwide. The incidence of prostate cancer ranks second in men. It is estimated that in 2022, there will be nearly 120,000 new cases of prostate cancer in mainland China, and the number in the United States will reach 260,000 [1, 2]. With the aging of the population, changes in people's living habits, and the promotion of PSA screening, the incidence of prostate cancer in China has increased significantly. As with other cancers, the success rate of treatment would greatly improve if prostate cancer patients could be diagnosed at an early stage.

Current detection and screening of prostate cancer rely extensively on serum prostate-specific antigen (PSA) screening, transrectal ultrasound (TRUS), and digital rectal examination (DRE) [3]. PSA testing is the mainstay of prostate cancer screening. However, whether PSA screening is suitable for the early diagnosis and treatment of PCa remains controversial. PSA screening leads to a small reduction in disease-specific mortality of prostate cancer within 10 years, but it does not affect overall mortality. Some data noted that the diagnostic sensitivity of prostate cancer decreases rapidly when the cutoff value of PSA was less than 4 ng/mL. There is no PSA cutoff point with high sensitivity and specificity for monitoring prostate cancer in men, which has led to the overtreatment of PSA [4–6] and places lingering physical and mental pressure on patients. Therefore, there is an urgent need for a new biomarker to meet the need for early detection of prostate cancer and evaluate the prognosis of prostate cancer.

Liquid biopsy, represented by cell-free DNA (cfDNA) and circulating tumor DNA (ctDNA), is gradually attracting the public's attention. Liquid biopsy uses blood, urine, feces, saliva, cerebrospinal fluid, pleural fluid, ascites, and semen as sample sources to obtain biomarkers using noninvasive means or minimally invasive surgery. It primarily includes circulating tumor cells (CTCs), cfDNA, cell-free RNA (cfRNA), extracellular vesicles, tumor-educated platelets (TEPs), and exosomes [7, 8]. There is abundant evidence that cfDNA plays an important role in early cancer detection, detection of recurrence, real-time monitoring of treatment, and detection of therapeutic targets. DNA methylation is one of the earliest, most stable, and most frequent alterations in the cancer genome. It has been extensively studied as a source of molecular biomarkers [9]. It is detected in cell-free DNA (cfDNA), and it is a valuable cancer biomarker. Glutathione-S-transferase p1 (GSTP1) is the most studied methylated biomarker, and it encodes glutathione-S-transferase, which is a detoxification enzyme and tumor suppressor involved in drug metabolism and protecting genes from oxidative stress [10]. Methylation of GSTP1 is frequently observed in prostate cancer tissue but is rare in normal prostate tissue, which suggests that methylated GSTP1 in cfDNA may serve as a biomarker for prostate cancer diagnosis.

Many studies demonstrated the potential diagnostic and prognostic value of cell-free DNA (cfDNA) as part of “liquid biopsy” in the treatment of prostate cancer. However, inconsistent conclusions remain in the literature due to differences in ethnicity, testing methods, sample types, and control sources. Therefore, we performed a meta-analysis of published clinical studies to assess the relationship between GSTP1 promoter methylation in cfDNA and the diagnosis and prognosis of prostate cancer. The present study provides a reference for the clinical application of GSTP1 methylation in prostate cancer.

## 2. Materials and Methods

### 2.1. Search Strategy

We performed this meta-analysis under the guidance of the PRISMA statement. We searched the PubMed, EMBASE, Web of Science, and PMC databases for English language literature published from the inception of the database to November 31, 2021. The search heading terms and keywords included “prostate cancer,” “GSTP1,” “cell free DNA,” and “methylation.” The references of the included articles were further searched to obtain other valuable sources.

### 2.2. Inclusion and Exclusion Criteria

Studies that met the following criteria were included as follows: (a) retrospective or prospective studies on the diagnostic and prognostic value of GSTP1 promoter methylation in prostate cancer published before November 31, 2021; (b) all PCa patients were pathologically diagnosed with prostate cancer after prostate biopsy or radical prostatectomy; (c) GSTP1 methylation was detected using PCR-based methylation assay in each study; (d) methylation detection data of the GSTP1 promoter on prostate cancer and control groups were provided, which may directly or indirectly calculate sensitivity, specificity, overall survival, and progression-free survival.

Studies meeting any of the following criteria were excluded as follows: (a) studies based on animal models or cell models; (b) reviews, case reports, conference papers, and letters; (c) duplicate studies; (d) studies consisting of fewer than five prostate cancer patients.

### 2.3. Data Extraction and Quality Assessment

According to a predesigned information extraction table, two investigators independently performed literature screening and data extraction to evaluate whether the titles and abstracts met the inclusion and exclusion criteria. If it was difficult to make a judgment based on the titles and abstracts, we checked the full text for verification. When the two researchers disagreed, a third party assisted in deciding whether to include the study, and any differences were resolved via group discussions. The extracted data included the following: (a) the first author, publication year, country or region of the patients, and treatment, (b) characteristics of participants (age, control type, methylation detection method, clinical and pathological stage, Gleason score, and the number of participants), (c) the incidence of GSTP1 promoter methylation in participants, and (4) sensitivity, specificity, and HRs for OS and PFS in PCa patients.

The Newcastle-Ottawa Scale (NOS) was used to evaluate the quality of the included case-control and cohort studies, including the three aspects of selection, comparability, and outcomes. The semiquantitative scoring principle was used, with a full score of 9 points. Higher scores indicate better quality. NOS scores of 5–9 were considered high-quality studies, and scores of 1–4 represented low-quality reports.

### 2.4. Statistical Analysis

STATA (version 15, USA) was used for data processing and meta-analysis. The collected relevant data from diagnostic studies were transformed into variables, such as true positive (TP), true negative (TN), false positive (FP), and false negative (FN). The data were further used to calculate pooled sensitivity, specificity, positive likelihood ratio (PLR), negative likelihood ratio (NLR), diagnostic odds ratio (DOR), area under the curve (AUC), and 95% confidence interval (95% CI). Hazard ratios and 95% CIs for OS and PFS were calculated for the prognostic studies. The heterogeneity of results among the included studies was assessed using the I^2^ statistic. If there was no statistical heterogeneity (*P* > 0.10 and *I*^2^ < 50%), the fixed-effect model was used for pooled analysis. Otherwise, the random-effect model was used for pooled analysis. Regression analyses were performed according to different clinical variables. Sensitivity analyses were performed in the presence of heterogeneity by excluding the included studies individually to examine whether specific omissions affected the stability of the overall results. If necessary, potential sources of heterogeneity were further investigated using stratified analysis. Deeks' funnel plots were used to evaluate the publication bias in diagnostic-related results, and Begg's test was used to assess the publication bias in prognostic-related results.

## 3. Results

### 3.1. Search Results

**Figure 1:**
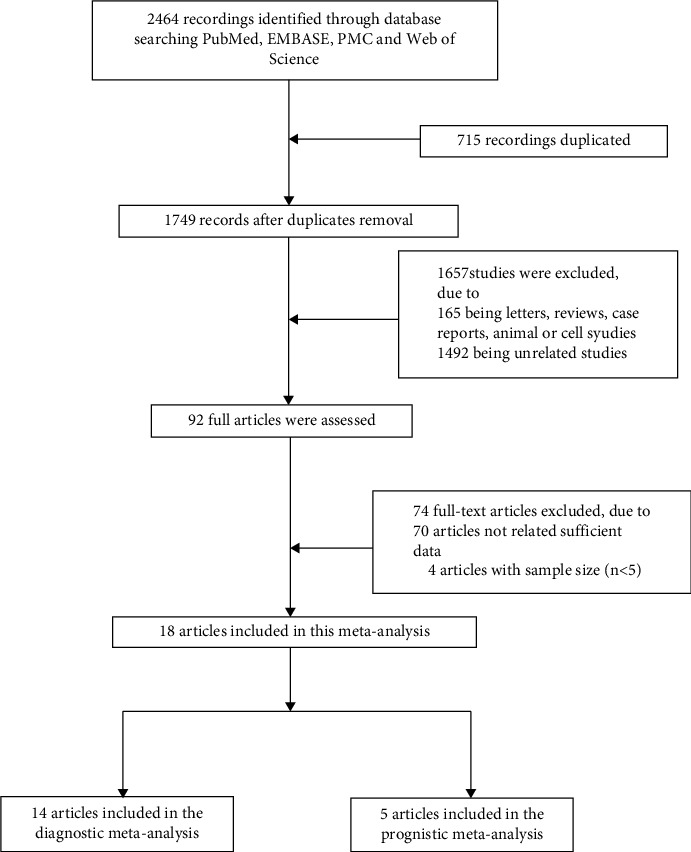
Flowchart of the study selection process in this meta-analysis.

The established search strategy retrieved 2464 relevant studies, and 1749 studies remained after the removal of duplicate reports. According to the inclusion and exclusion criteria, 1657 studies that did not meet the requirements were removed, and 92 studies were initially included. A further 74 studies were excluded due to a lack of sufficient data (*n* = 70) and a low number of samples (*n* = 4). Eighteen studies were ultimately included, of which 77.8% (14/18) were related to diagnostic meta-analysis and 27.8% (5/18) were related to prognostic meta-analysis. A total of 2610 samples were included in this study. The diagnostic meta-analysis included 900 patients with prostate cancer and 697 controls, and the prognostic meta-analysis included 964 patients with prostate cancer and 81 controls. The control group was primarily composed of patients with benign prostatic hyperplasia (BPH), healthy volunteers, and patients with negative prostate biopsy. The studies were published from 2001 to 2019, and most participants were from Europe, with the rest being from North and South America. A flowchart of the search is shown in Figure 1, and the basic characteristics of the 18 included studies are summarized in Table 1.

### 3.2. Quality Assessment

The Newcastle-Ottawa Scale (NOS) was used to evaluate the quality of 18 included studies, with a full score of 9 points. A NOS score of 5–9 was considered high-quality research. The 18 studies in this meta-analysis scored 6–8 points, with an average score of 7 points. This result indicated that the overall quality of the included literature was good (Table S1).

### 3.3. Meta-Analysis Results Related to Diagnosis

**Figure 2:**
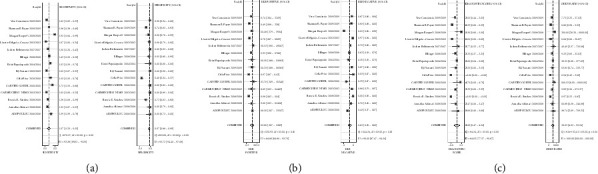
Diagnostic value of GSTP1 methylation in PCa. (a) Forest plots for sensitivity and specificity. (b) Forest plots for PLR and NLR. (c) Forest plots for DOR. (PLR: positive likelihood ratio; NLR: negative likelihood ratio; DOR: diagnostic odds ratio).

**Figure 3:**
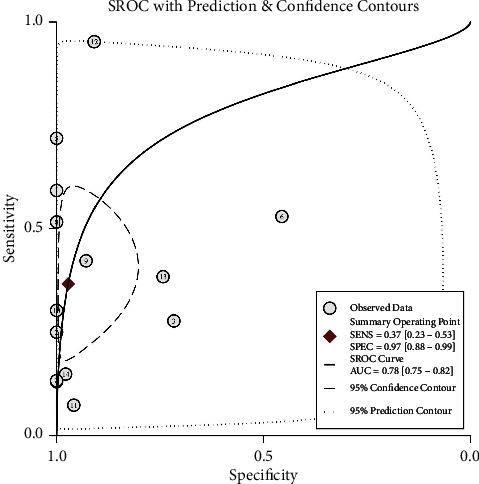
SROC curves of GSTP1 methylation in cfDNA for the diagnosis of PCa.

Differences in GSTP1 promoter methylation in plasma cfDNA between prostate cancer patients and controls were compared. Sensitivity (SEN), specificity (SPE), positive likelihood ratio (PLR), negative likelihood ratio (NLR), and diagnostic odds ratio (DOR) were used as effect sizes to analyze the heterogeneity of the study. There was significant heterogeneity (*P* < 0.001, *I*^2^ = 92.38%; *P* < 0.001, *I*^2^ = 95.75%; *P* < 0.001, *I*^2^ = 84.80%; *P* < 0.001, *I*^2^ = 91.01%; *P* < 0.001, *I*^2^ = 100%), and random-effect models were used. The results showed that the combined sensitivity of cfDNA in the diagnosis of prostate cancer was 0.37 (95% CI = 0.23, 0.53), specificity combined 0.97 (95% CI = 0.88, 0.99), PLR combined 13.11 (95% CI = 3.07, 55.89), NLR combined 0.65 (95% CI = 0.51, 0.83), and DOR combined 20.12 (95% CI = 4.33, 93.54) (Figure 2). These results showed that the proportion of GSTP1 methylation in PCa patients was higher than controls, and the difference was statistically significant (*P* < 0.01). To evaluate the diagnostic accuracy and utility of GSTP1 methylation as a molecular marker for prostate cancer, we constructed a summary receiver operating characteristic (SROC) curve (Figure 3). The corresponding SROC curve showed that the pooled sensitivity and specificity were 37% and 97%, respectively, and the area under the curve was 0.78 (95% CI = 0.75, 0.82). GSTP1 methylation in cfDNA discriminated between PCa and controls individuals with relatively high specificity.

### 3.4. Meta-Analysis Results Related to Prognosis

**Figure 4:**
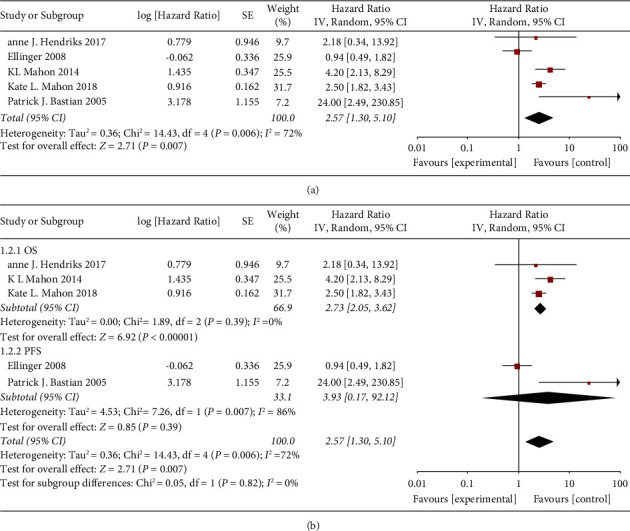
Prognostic value of GSTP1 methylation in prostate cancer. (a) Forest plots for overall prognostic analysis. (b) Forest plots for the survival index analysis subgroup.

We explored the relationship between PCa prognosis and methylation. The heterogeneity test of the five studies related to prognosis (*I*^2^ = 72% > 50%, and *P*=0.006 < 0.1) (Figure 4(a)) indicated strong heterogeneity among the results, and a random effect model was selected for meta-analysis. Based on the data, it was highly suspected that the source of heterogeneity was the inconsistency of observation indicators, and subgroup analysis was subsequently performed. The results of the meta-analysis showed that the hypermethylation of GSTP1 was associated with shorter survival in PCa patients (HR = 2.57, 95% CI = 1.30, 5.10) (Figure 4(a)), which was statistically significant (*Z* = 2.71, *P* < 0.05).

### 3.5. Sensitivity Analysis

We used sensitivity analysis to verify the effect of each study on the overall results by randomly removing individual studies. The results showed that removal of any one of the included studies did not significantly affect the overall estimate. This result indicated that the results of this meta-analysis were stable and reliable (Figure S1).

### 3.6. Regression and Subgroup Analysis

We also performed regression analysis for diagnosis-related results to further examine potential sources of heterogeneity. The regression analysis included variables such as region (European or not), sample type (plasma or not), and detection method (MSP or not). The results of regression analysis suggested that the variables of region and detection method were responsible for the heterogeneity (Figure S2).

Because the heterogeneity test found that the studies related to prognosis had a high degree of heterogeneity, the observation index (OS/PFS) was used as the stratification standard to explore the source of heterogeneity. Five studies related to prognosis were divided into two groups for meta-analysis (Figure 4(b)). Subgroup analysis showed that using OS as the observation index had lower heterogeneity (*I*^2^ = 0%, *P*=0.39) than using PFS as the observation index (*I*^2^ = 86%, *P*=0.007). The HR value of the PFS subgroup was higher than the OS subgroup, but the difference was not statistically significant (*P*=0.82). The results of subgroup analysis indicated that the heterogeneity of the studies may be due to the differences in observation indicators.

### 3.7. Fagan Plot

We also drew a Fagan plot and set the pretest probability to 56%. The results showed that a positive GSTP1 methylation test was 94% accurate in diagnosing prostate cancer, and a negative test was 45% accurate. This result suggested that GSTP1 methylation in cfDNA had good accuracy in diagnosing prostate cancer (Figure S3).

### 3.8. Publication Bias

**Figure 5:**
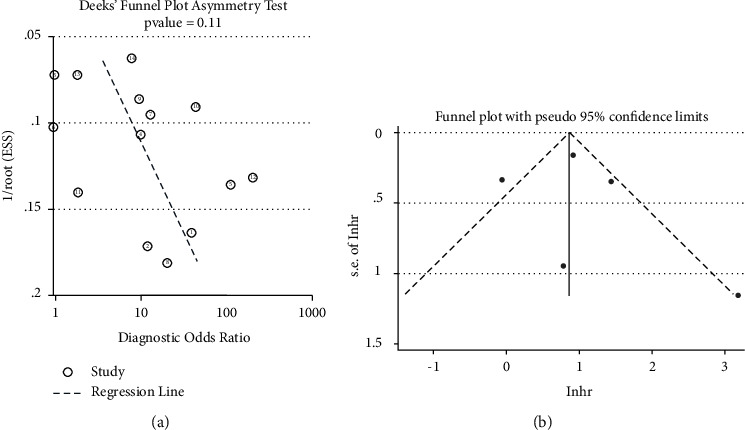
The assessment of potential publication bias in the meta-analysis. (a) Deeks' test for the diagnostic meta-analysis. (b) Begg's test for the overall survival meta-analysis.

A funnel plot was used to test the potential publication bias of the studies related to diagnosis and prognosis. Deeks' funnel plot showed no obvious publication bias in the diagnostic experiment (*P*=0.11, Figure 5(a)), which suggests that the results of the diagnostic meta-analysis were reliable and robust. There was no evidence showing publication bias in the prognostic meta-analysis according to Begg's test (*P*=0.462, Figure 5(b)), which suggests that the results were equally reliable.

## 4. Discussion

Cell-free DNA (cfDNA) is generally derived from normal or tumor cells. CfDNA is released via apoptosis, necrosis, and active secretion and transported into the bloodstream [29]. CfDNA is a double-stranded fragment with a length of approximately 150 to 200 bp [30]. Many studies reported promoter methylation in cfDNA, and methylation of the GSTP1 promoter is the most promising. Glutathione-S-transferase (GST) is a key enzyme involved in DNA protection from electrophilic metabolites of carcinogens and reactive oxygen species by conjugating chemically reactive electrophiles to glutathione [31]. Aberrant methylation of the GSTP1 promoter is not limited to prostate cancer, and it occurs in other cancers, including lung cancer [32] and breast cancer [33]. Significant differences in the length of cfDNAs from tumor and nontumor cells may allow for the identification of cancer-derived fragments, which makes it more accurate to distinguish tumors from benign diseases than other methods [7, 34].

Currently, PSA is considered to be the most important marker for prostate cancer screening. It has been reported that PSA has a sensitivity of up to 91% but a specificity of only 14% for the diagnosis of PCa when the cut-off value is 2.5 ng/mL [35]. The detection of GSTP1 promoter methylation has a high specificity in our study [0.97 (95% CI = 0.88, 0.99)], which is significantly higher than PSA testing. This partly compensates for the lack of specificity in early PSA screening for prostate cancer. Therefore, GSTP1 methylation can be combined with PSA screening to improve the diagnostic specificity of PCa. However, its sensitivity is not higher that the traditional biomarker. The sensitivity of 14 diagnostic studies ranged from 0.07 to 0.95. Due to the different detection methods and regions, the sensitivity of various studies is highly heterogeneous. These results suggest that neither cfDNA nor PSA alone are accurate enough to screen for prostate cancer. Bastian et al. [36] proposed that the combination of multiple DNA methylation achieved higher sensitivity and specificity than a single GSTP1 methylation. Many recent studies also showed that the detection of GSTP1 promoter methylation in plasma, serum, or urine samples in combination with PSA screening significantly improved the diagnostic accuracy of PCa [37]. Therefore, our data suggest that methylation of GSTP1 in plasma or serum samples is more suitable as a complement to serum PSA testing rather than a complete replacement.

The prognosis of PCa patients is generally different. If the recurrence risk of patients can be differentiated and the response of the tumor to the drug can be evaluated, patient's survival will be greatly prolonged. The half-life of cfDNA ranges from minutes to several hours, which is much shorter than protein-based biomarkers, such as PSA, which takes several weeks to undergo changes representative of tumor dynamics [38, 39]. Liquid biopsy, represented by cfDNA, tracks changes in cfDNA during cancer treatment to closely monitor tumor behavior and response to treatment. Liquid biopsy aids in the assessment of prognosis, primary and acquired drug resistance, and disease surveillance in advanced diseases. The results of this meta-analysis suggest that GSTP1 promoter methylation in cfDNA predicts disease progression and a high risk of death in PCa patients. A recently published phase III multicenter trial including 600 CRPC patients showed that detectable serum methylated GSTP1 levels before and after two cycles of chemotherapy were independently associated with shorter overall survival. Undetectable serum methylated GSTP1 was associated with a longer time to PSA progression after two cycles of docetaxel treatment [12]. Further studies reported that GSTP1 methylation combined with other frequently methylated genes may be more helpful in assessing the prognosis of PCa patients, especially in mCRPC patients, such as the methylation of GSTP1 and RASSF2A [19] and the methylation of GSTP1, RASSF1, and RARB [18].

The innovation of this meta-analysis lies in the comprehensive analysis of the value of GSTP1 promoter methylation in the diagnosis and prognosis of PCa, using cfDNA-based liquid biopsy as an entry point. However, there are still some deficiencies in the study due to the limitations of the meta-analysis itself. For example, there were few prognosis-related studies included in this study, which had certain heterogeneity. There were some important confounding factors that could not be controlled in the experiments, and the final results may be biased due to the differences in sample collection time and detection equipment. Because some of the original studies did not provide data on tumor stage, PSA level, Gleason score, age, and treatment, a comprehensive subgroup analysis was not performed. We tried to obtain the PSA and GSTP1 methylation ratio data of patients to verify the combination value of the two in the diagnosis and prognosis of PCa, but we did not obtain relevant data in the supplementary materials. Notably, we found that most studies in this field were small-scalecase-control studies, and randomized controlled studies to provide evidence were lacking. Therefore, it must be validated in a larger independent cohort. The research population in this field is primarily concentrated in the Caucasian race, and there is a lack of research in different ethnic groups, especially in Asian populations. Therefore, more ethnic groups must be studied to improve the research results.

## 5. Conclusions

In conclusion, our meta-analysis demonstrated that GSTP1 promoter methylation in cfDNA may be used as a potential biomarker for prostate cancer and has high clinical value for the diagnosis and prognosis of PCa. It should be noted that the sensitivity of GSTP1 methylation as a separate screening indicator remains limited. The combination of GSTP1 methylation with traditional PSA screening or methylation of multiple genes may produce better results than either of the method alone. Liquid biopsies centered on GSTP1 methylation in cfDNA will provide a reliable biomarker for patients with PCa.

## Figures and Tables

**Table 1:** Baseline characteristics of the 18 eligible articles.

Cations	Authors	Year	Country	Patients type (cancer/control)	Number (cancer/control)	TP/FP/TN/FN	Detection methods	Meta-type
[11]	Constancio et al.	2019	Portugal	PCa/health	121/136	18/3/133/103	Q-MSP	Dia
[12]	Mahon et al.	2019	Australia	mCRPC/NA	562/NA	NA	MSP	OS
[13]	Sanchez et al.	2018	Mexico	PCa/BPH	83/113	23/32/81/60	Q-MSP	Dia
[14]	Hendriks et al.	2018	Netherlands	PCa/health	47/30	NA	MSP	OS
[15]	Mahon et al.	2014	Australia	CRPC/NA	126/NA	NA	MSP	OS
[16]	Delgado-Cruzata et al.	2012	USA	PCa/health	27/24	2/1/23/25	Q-MSP	Dia
[17]	Prior et al.	2010	Spain	PCa/health	34/79	18/43/36/16	MSP	Dia
[18]	Sunami et al.	2009	Germany	PCa/health	83/40	11/0/40/72	MSP	Dia
[19]	Payne et al.	2009	USA	PCa/health	91/101	35/26/75/56	BS	Dia
[20]	Altimari et al.	2008	Germany	PCa/BPH + health	168/42	71/3/39/97	MSP	Dia
[21]	Ellinger et al.	2008	Italy	PCa/health	16/16	4/0/16/12	Q-MSP	Dia, PFS
[22]	Roupret et al.	2008	UK	PCa/health	42/22	40/2/20/2	Q-MSP	Dia
[23]	Reibenwein et al.	2007	Austria	PCa/health	76/49	23/0/49/53	MSP	Dia
[24]	Papadopoulou et al.	2006	Greece	PCa/BPH	27/13	16/0/13/11	MSP	Dia
[25]	Bastian et al.	2005	USA	PCa/health	213/35	NA	Q-MSP	PFS
[26]	Papadopoulo et al.	2004	Greece	PCa/health	31/9	16/0/9/15	MSP	Dia
[27]	Jernimo et al.	2002	Portugal	PCa/BPH	69/31	9/0/31/60	MSP	Dia
[28]	Goessl et al.	2001	Germany	PCa/BPH	32/22	23/0/22/9	MSP	Dia

Note: MS-PCR = methylation-specific PCR; qMS-PCR = quantitativemethylation-sensitive PCR; BS = bisulphite sequencing; NA = not available; TP = true positive; TN = true negative; FP = false positive; FN = false negative; PCa = prostate cancer; BPH = benign prostatic hyperplasia; mCRPC = metastatic castration-resistant prostate cancer; Dia = diagnostic design; OS = overall survival; PFS = progression free survival.

## Data Availability

The data that support the findings of this study are available in the supplementary material of this article.

## Supplementary Materials

Supplementary Materials Table S1: the Newcastle-Ottawa Scale (NOS) to assess the quality of the case-control studies. Figure S1: sensitivity analysis of GSTP1 methylation in cfDNA for the diagnosis of PCa. Figure S2: forest plots of multivariable meta-regression for SEN and SPE. Figure S3: the Fagan plot showing the diagnostic value of GSTP1 methylation in cfDNA for prostate cancer.
