# Supplementary material for: Glutathione-S-Transferase p1 Gene Promoter Methylation in Cell-Free DNA as a Diagnostic and Prognostic Tool for Prostate Cancer: A Systematic Review and Meta-Analysis
Source: Int J Endocrinol. 2023 Jan 28;2023:7279243. doi: 10.1155/2023/7279243 (PMC9899149; doi:10.1155/2023/7279243)

**Supplementary Materials**

These supplementary materials were for the article entitled

“**Glutathione-S-transferase p1 gene promoter methylation in cell-free DNA as a diagnostic and prognostic tool for prostate cancer: A systematic review and meta-analysis**”.

**Figure Legends**

Table S1: The Newcastle–Ottawa Scale (NOS) to assess the quality of the case–control studies

Figure S1: Sensitivity analysis of GSTP1 methylation in cfDNA for the diagnosis of PCa

Figure S2: Forest plots of multivariable meta-regression for SEN and SPE

Figure S3: The Fagan plot showing the diagnostic value of GSTP1 methylation in cfDNA for prostate cancer.

Table S1: The Newcastle–Ottawa Scale (NOS) to assess the quality of the case–control studies

| Studies |  | Selection |  |  |  | Comparability |  |  |  | Exposure |  | Total score |
| --- | --- | --- | --- | --- | --- | --- | --- | --- | --- | --- | --- | --- |
| Authors (year) | Is the case definition adequate? | Representa- tiveness of the cases | Selection of controls |  | Definition of controls | Studies controlling the most important factors | Studies control- ling the other main factors |  | Ascertainment of exposure | Same method of ascertainment for cases and controls | Non-Response  rate |  |
| Vera et al.(2019) | * | * |  |  | * | * | * |  | * | * |  | 7 |
| Mahon et al. (2019) | * | * | * |  | * | * | * |  | * | * |  | 8 |
| Beatriz et al. (2018) | * | * |  |  | * | * | * |  | * | * |  | 7 |
| Hendriks et al. (2018) | * | * | * |  | * | * | * |  |  | * |  | 7 |
| K L Mahon et al.(2014) | * | * | * |  | * | * | * |  | * | * |  | 8 |
| Lissette et al. (2012) | * | * |  |  | * | * | * |  | * | * |  | 7 |
| Celia et al.(2010) | * | * |  |  | * | * | * |  | * | * |  | 7 |
| Eiji et al.(2009) | * | * | * |  | * | * | * |  | * | * |  | 8 |
| Shannon et al.(2009) | * | * |  |  | * | * | * |  | * | * |  | 7 |
| Annalisa et al. (2008) | * | * |  |  | * | * | * |  | * | * |  | 7 |
| Ellinger et al.(2008) | * | * |  |  | * | * | * |  | * | * |  | 7 |
| Morgan et al.(2008) | * |  | * |  | * | * | * |  | * | * |  | 7 |
| Jochen et al.(2007) | * | * |  |  | * | * | * |  | * | * |  | 7 |
| ADOPOULOU et al.(2006) | * | * |  |  | * | * | * |  | * | * |  | 7 |
| Patrick J.et al.(2005) | * | * | * |  | * | * | * |  | * |  |  | 7 |
| Papadopoulo et l.(2004) | * | * |  |  | * |  | * |  | * | * |  | 6 |
| CARMEN et al.(2002) | * | * |  |  | * | * | * |  | * | * |  | 7 |
| GOESSL et al.(2001) | * | * |  |  | * | * | * |  | * | * |  | 7 |

Figure S1: Sensitivity analysis of GSTP1 methylation in cfDNA for the diagnosis of PCa


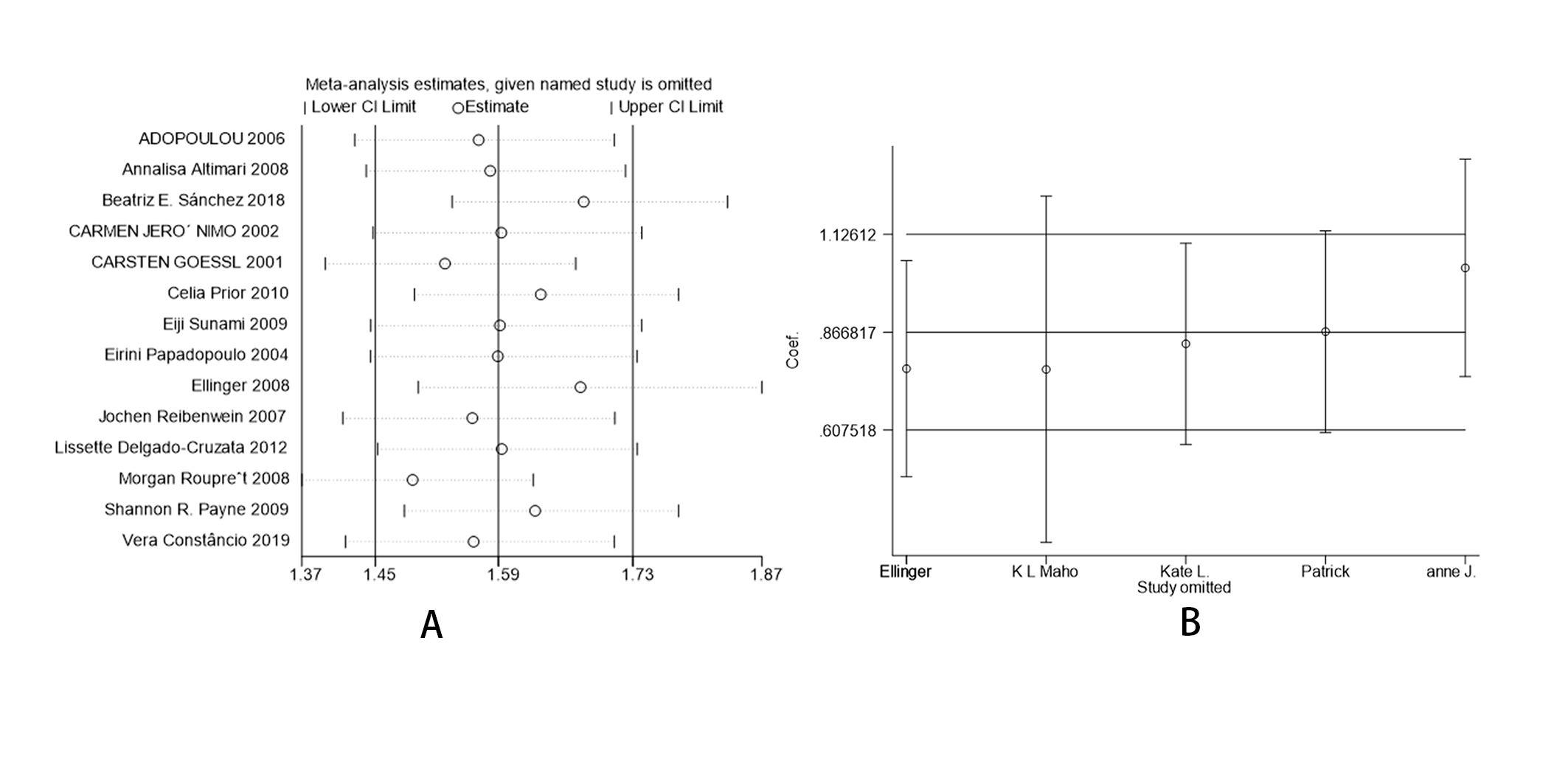


Figure S2: Forest plots of multivariable meta-regression for SEN and SPE


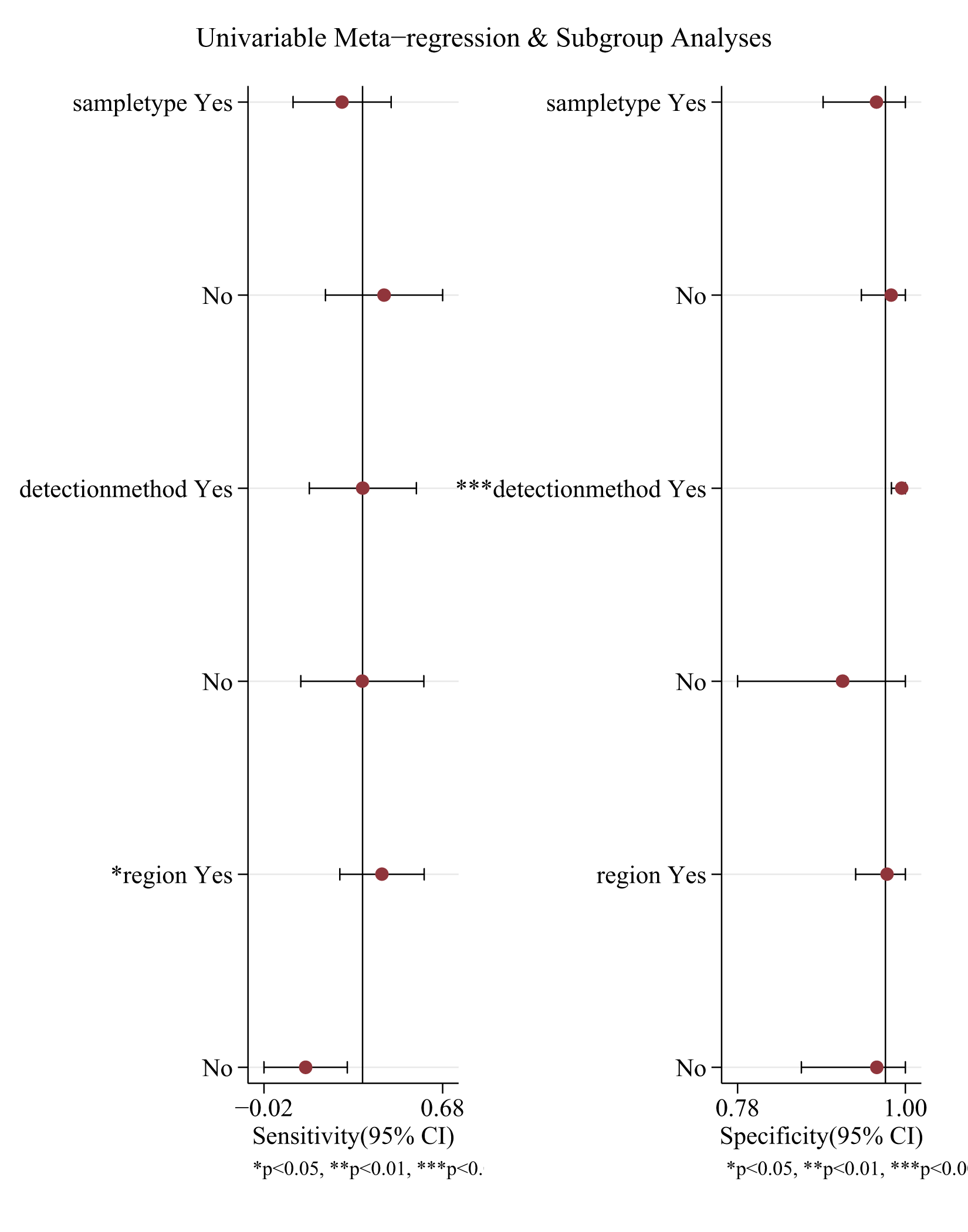


Figure S3: The Fagan plot showing the diagnostic value of GSTP1 methylation in cfDNA for prostate cancer.


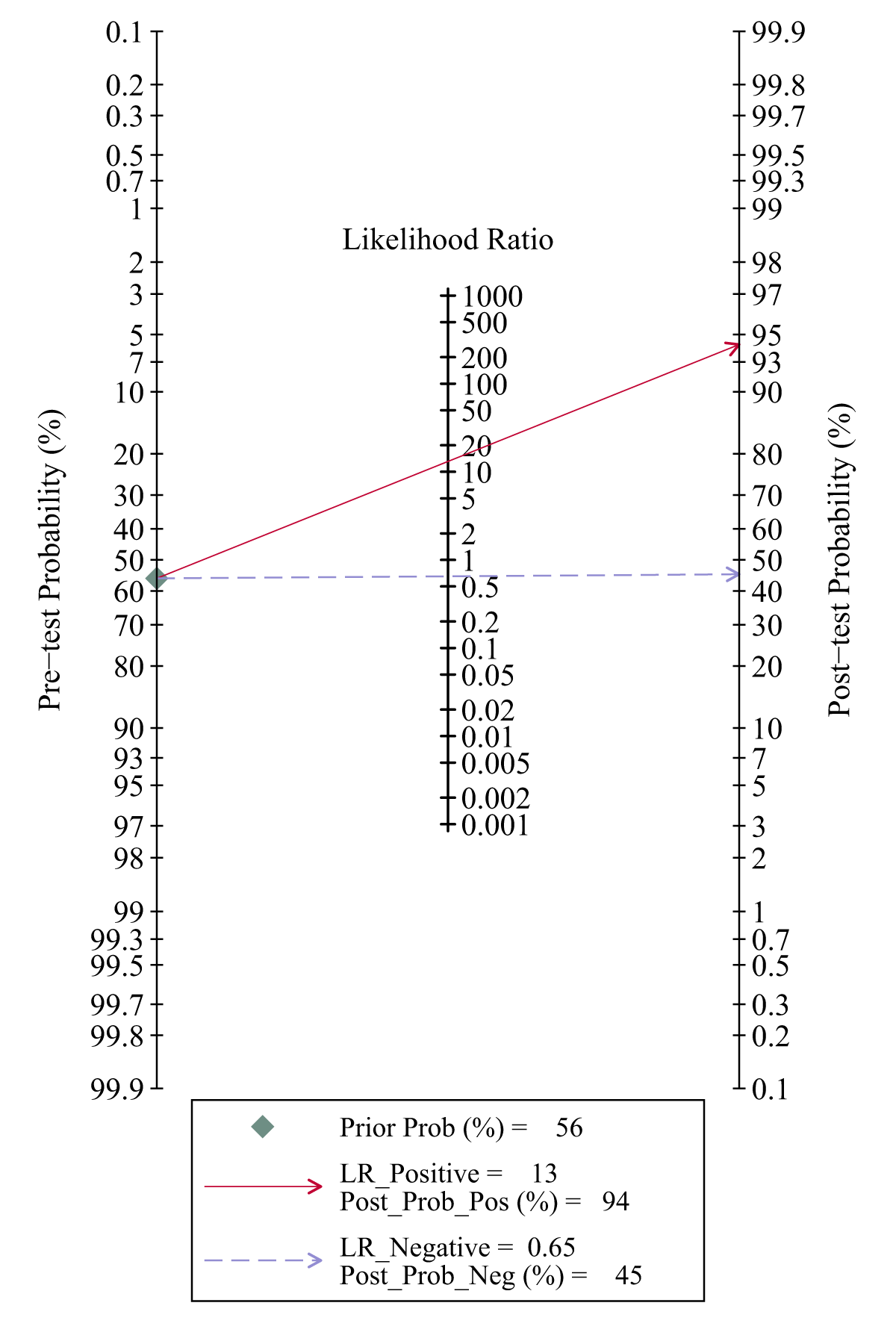

Supplement: Supplementary Materials — Table S1: the Newcastle-Ottawa Scale (NOS) to assess the quality of the case-control studies. Figure S1: sensitivity analysis of GSTP1 methylation in cfDNA for the diagnosis of PCa. Figure S2: forest plots of multivariable meta-regression for SEN and SPE. Figure S3: the Fagan plot showing the diagnostic value of GSTP1 methylation in cfDNA for prostate cancer. [file 7279243.f1.docx]
